# MARCH2: Comparative Assessment of Therapeutic Effects of Acarbose and Metformin in Newly Diagnosed Type 2 Diabetes Patients

**DOI:** 10.1371/journal.pone.0105698

**Published:** 2014-08-22

**Authors:** Guang Wang, Jia Liu, Ning Yang, Xia Gao, Hui Fan, Yuan Xu, Wenying Yang

**Affiliations:** 1 Department of Endocrinology, Beijing Chaoyang Hospital Affiliated to Capital Medical University, Beijing, P. R. China; 2 Department of Endocrinology, China-Japan Friendship Hospital, Beijing, P. R. China; University of Warwick – Medical School, United Kingdom

## Abstract

**Background:**

The data of MARCH (Metformin and AcaRbose in Chinese as the initial Hypoglycaemic treatment) trial demonstrated that acarbose and metformin have similar efficacy as initial therapy for hemoglobin A1c (HbA1c) reduction in Chinese patients with newly diagnosed type 2 diabetes. We investigated whether the therapeutic efficacy was diversified under different body mass index (BMI) status.

**Methods:**

All 784 subjects were divided into normal-weight group (BMI<24 kg/m^2^), overweight group (BMI 24–28 kg/m^2^) and obese group (BMI≥28 kg/m^2^). Patients were assigned to 48 weeks of therapy with acarbose or metformin, respectively. The clinical trial registry number was ChiCTR-TRC-08000231.

**Results:**

The reduction of HbA1c levels and the proportion of patients with HbA1c of 6.5% or less were similar in the three groups after acarbose and metformin treatment. In overweight group, fasting blood glucose (FBG) after metformin treatment showed greater decline compared to acarbose group at 48 weeks [−1.73 (−1.99 to −1.46) vs. −1.37 (−1.61 to −1.12), *P*<0.05), however the decrease of 2 h post-challenge blood glucose (PBG) after acarbose treatment at 48 weeks was bigger compared to metformin group [−3.34 (−3.83 to−2.84) vs. −2.35 (−2.85 to −1.85), *P*<0.01 ]. Both acarbose and metformin treatment resulted in a significant decrease in waist circumference, hip circumference, weight and BMI in the three groups (all *P*<0.05).

**Conclusion:**

Acarbose and metformin decreased HbA1c levels similarly regardless of BMI status of Chinese type 2 diabetic patients. Acarbose and metformin resulted in a significant and modest improvement of anthropometric parametres in different BMI status. Thus, acarbose treatment may contribute a similar effect on plasma glucose control compared to metformin, even in obesity patients.

**Trial Registration:**

ChiCTR.org ChiCTR-TRC-08000231

## Introduction

Type 2 diabetes is a metabolic disease with high mortality and morbidity [1]. For treating diabetes, numerous novel glucose-lowering agents have been developed [2], however, which are more expensive and lack long-term follow-up safety data. As the classical hypoglycemic drugs [3], acarbose and metformin have lower cost, and long-term safety has been confirmed in more than hundreds of studies [4]–[5].

The result of the MARCH trial demonstrates that acarbose and metformin have similar efficacy on lowering HbA1c as initial therapy for newly diagnosed type 2 diabetic patients in China [6]. However, it is unclear whether the above mentioned therapeutic efficacy was diversified under the different BMI status, such as in normal weight, overweight and obese patients. In the present study, we assessed the therapeutic effect of acarbose and metformin in newly diagnosed type 2 diabetic patients with different BMI status by reanalyzing data from the MARCH trial.

## Materials and Methods

### Design and participants

We analyzed the data from MARCH, a randomized, open-label, non-inferiority trial designed to compare acarbose with metformin as the initial therapy in Chinese patients newly diagnosed with type 2 diabetes. This study was registered with Chinese Clinical Trial Registry, number ChiCTR-TRC-08000231. The protocol was approved by the Ethics Committee from each clinical site (China-Japan Friendship Hospital, Beijing, China; Shanxi Province People's Hospital, Taiyuan, China; The First Hospital of China Medical University, Shenyang, China; West China Hospital, Sichuan University, Chengdu, China; Xiangya Second Hospital of Central South University, Changsha, China; Xijing Hospital, Fourth Military Medical University, Xi'an, China; The Third Affiliated Hospital of Sun Yatsen University, Guangzhou, China; Shanghai jiaotong University Affiliated Sixth People's Hospital, Shanghai, China; Chinese People's Liberation Army General Hospital, Beijing, China; Gansu Provincial Hospital, Lanzhou, China; Peking Union Medical College Hospital, Beijing, China; Beijing Chaoyang Hospital Affiliated to Capital Medical University, Beijing, China) [6]. 784 Patients [HbA1c between 7% and 10%; fasting blood glucose (FBG) between 7.0 mmol/L and 11.1 mmol/L] were recruited from 11 centers, and were diagnosed as type 2 diabetes within the past 12 months according to WHO diabetes criteria in 1999. They had either not taken anti-diabetic drugs or been on short-term (1 month) treatment that had been stopped for 3 months. None of the patients had a history of unstable angina, acute myocardial infarction, liver function impairment, renal function impairment, haematological diseases, chronic hypoxic diseases (emphysema and cor pulmonale), intestinal surgery and infectious disease.

After 4 weeks run-in phase, patients randomly received metformin hydrochloride (1500 mg/d) or acarbose (300 mg/d), with 24-week monotherapy and 24-week add-on therapy with insulin secretagogues if needed. Bayer Healthcare (China) provided acarbose, and Double Crane Phama provided metformin. According to 2007 Chinese management guideline, add-on therapy with insulin secretagogues was began at 24 weeks when the HbA1c was higher than 7%, or FBG was higher than 7 mmol/L. After 24-weeks monotherapy, five patients in acarbose group and three patients in metformin group received insulin secretagogues. All participants gave written informed consent.

### Measurements

At baselines, all patients underwent a clinical assessment including bodyweight, waist circumference, hip circumference, oral glucose tolerance test [FBG and 2 h post-challenge blood glucose (PBG)], fasting serum insulin (FINS), lipid profile [triglycerides (TG), total cholesterol (TC), low-density lipoprotein cholesterol (LDL-C) and high-density lipoprotein cholesterol (HDL-C) ] and HbA1c. A detailed medical history was recorded for previous concomitant diseases and medication status. Anthropometric and biochemical measurements were repeated at 24 weeks and 48 weeks. Non-HDL-C level was calculated using the equation: non-HDL-C (mmol/L)  = TC – HDL-C [7]. We evaluated the proportion of patients with optimal levels of LDL-C and non-HDL-C. The optimal levels of LDL-C and non-HDL-C were defined as: LDL-C<100 mg/dL (2.6 mmol/L) and non-HDL-C<130 mg/dL (2.6 mmol/L) [8]. We calculated homeostasis model assessment of insulin resistance (HOMA-IR) and homeostasis model assessment of β cell function (HOMA-β) by using the following equation: HOMA-IR  =  [FBG (mmol/L)* FINS (mIU/L) /22.5]; HOMA-β = 20*FINS (mIU/L) / [FBG (mmol/L) - 3.5] [9]–[10]. According to BMI value, these subjects were divided into three groups: normal weight group (<24 kg/m^2^), overweight group (24–28 kg/m^2^) and obese group (≥28 kg/m^2^) [11]. We compared the changes of metabolic parameters under different BMI status.

### Statistical methods

Data were analyzed using SPSS 17.0 (SPSS, Inc, Chicago, IL). Continuous data were expressed as means ±SD. Because TG, FINS, HOMA-IR and HOMA-β did not follow a normal distribution, the values were given as medians, the upper and lower quartiles. Changes in parameters from the baseline values within group were evaluated using two-tails paired t-test. The differences between groups were analyzed by ANOVA test. Comparision between groups at baseline and after treatment was done with independent sample t-test. The differences of proportions were analyzed by chi-square test. Statistical significance was inferred when *P*<0.05.

## Results

### Baseline of characteristics in type 2 diabetic patients

Table 1 presents baseline of characteristics in normal weight, overweight and obese groups. With increased BMI of patients, the prevalences of hypertension and non-alcoholic fatty liver disease got higher (all *P*<0.05). Systolic blood pressure, TC, LDL-C, FBG and HbA1c were comparable in the three groups (all *P*>0.05). A significant trend was presented for age, waist circumference, hip circumference, body weight, diastolic blood pressure, HDL-C, TG, Non-HDL-C, PBG, FINS, HOMA-IR, HOMA-β, and the proportion of patients with optimal levels of LDL-C and non-HDL-C among all groups (all *P*<0.05). In comparisons of variables between acarbose and metformin arms of the three groups, all parameters were similar except for FINS, HOMA-IR and HOMA-β in overweight group (*P*<0.05).

**Table 1:** Baseline characteristics of normal weight, overweight and obese patients with newly diagnosed type 2 diabetes.

Parameters	BMI<24	BMI 24–28	BMI>28	*P*
	(n = 216)	(n = 405)	(n = 163)	
Age,y	51.57±9.69	50.83±9.12	48.34±8.68	.002
Gender, Males/Females, n	108/108	252/153	104/59	.872
Dyslipidemia, n (%)	123(56.9)	275(67.9)	126(77.3)	.074
Hypertension, n (%)	41(19.0)	112(27.7)	69(42.3)	.000
NAFLD, n (%)	9(4.2)	19(4.7)	20(12.3)	.000
CHD, n (%)	3(1.4)	11(2.7)	3(1.8)	.124
Waist circumference, cm	81.95±6.69	90.20±6.25	97.20±6.54	.000
Hip circumference, cm	92.65±5.73	99.32±6.22	105.40±5.77	.000
Weight, kg	60.12±7.38	70.92±7.75	80.85±8.48	.000
BMI, kg/m^2^	22.42±1.23	25.92±1.06	29.19±0.60	.000
Systolic BP, mm Hg	121.87±12.52	124.41±13.47	124.22±12.63	.057
Diastolic BP, mm Hg	76.62±7.75	79.40±8.48	81.64±8.83	.000
TC, mmol/L	5.13±1.16	5.29±1.15	5.33±1.01	.157
LDL-C, mmol/L	2.98±0.93	3.09±0.93	3.07±0.81	.365
HDL-C, mmol/L	1.28±0.35	1.23±0.29	1.19±0.25	.013
TG, mmol/L	1.64 (1.20–2.34)	1.88(1.28–2.56)	2.17 (1.45–3.10)	.035
Non-HDL-C, mmol/L	3.84±1.12	4.05±1.12	4.14±1.01	.019
FBG, mmol/L	8.53±1.66	8.30±1.46	8.22±1.38	.101
PBG, mmol/L	13.22±3.32	12.57±2.89	11.97±2.65	.000
FINS, uIU/mL	8.85 (5.30–13.51)	10.87 (7.18–16.04)	15.59 (10.96–20.52)	.000
optimal LDL-C rate, %	35.6	28.1	27.0	.000
optimal non-HDL-C rate, %	35.6	27.9	22.7	.000
HbA1c, %	7.60±1.39	7.56±1.19	7.42±1.07	.340
HOMA-IR	3.27 (1.98–5.32)	3.92 (2.48–6.14)	5.56 (3.73–7.39)	.000
HOMA-β	37.81 (21.74–62.17)	47.54 (28.93–74.14)	66.44 (49.40–94.27)	.000
Fibrates, n (%)	2(0.9)	12(3.0)	5(3.1)	.179
Statins, n (%)	9(4.2)	26(6.4)	12(7.4)	.138

Data are means ±SD unless indicated otherwise. TG, INS, HOMA-IR and HOMA-β are shown as median and range. NAFLD: non-alcoholic fatty liver disease; CHD: coronary heart disease. BMI: body mass index; BP: blood pressure; TC: total cholesterol; LDL-C: low-density lipoprotein cholesterol; HDL-C: high-density lipoprotein cholesterol; TG: triglyceride; FBG: fasting blood glucose; PBG: 2 h post-challenge blood glucose; FINS: fasting insulin; HbA1c: hemoglobin A1c; HOMA-IR: homeostasis model assessment of insulin resistance; HOMA-β: homeostasis model assessment of β cell function; optimal LDL-C rate: the proportion of patients with optimal levels of LDL-C; optimal non-HDL-C rate: the proportion of patients with optimal levels of non-HDL-C. The optimal levels of LDL-C and non-HDL-C were defined as: LDL-C<100 mg/dL (2.6 mmol/L) and non-HDL-C<130 mg/dL (2.6 mmol/L) [7].

### The changes in parameters of glucose metabolism after acarbose or metformin treatment

Both acarbose and metformin treatment significantly decreased HbA1c levels at 24 weeks and 48 weeks in the three groups (all *P*<0.05) (table 2), respectively. Our previous study has shown that there was no difference in the proportion of patients with HbA1c of 6.5% or less between acarbose and metormin at 24weeks and 48weeks [6]. Interestingly, in the three groups, the proportion of patients with HbA1c of 6.5% or less was similar after 24 weeks and 48 weeks of metformin or acarbose treatment (all *P*>0.05) (table 2). The significant reductions in FBG, PBG, and FINS were observed in the three groups with acarbose or metformin treatment for 24 weeks and 48 weeks (all *P*<0.05). In overweight groups, FBG after metformin treatment showed greater decline compared to acarbose treatment group at 48 weeks [−1.73 (−1.99 to −1.46) vs. −1.37 (−1.61 to −1.12), *P*<0.05)], however the decrease of PBG after acarbose treatment for 48 weeks was more than metformin group [−3.34 (−3.83 to−2.84) vs. −2.35 (−2.85 to −1.85), *P*<0.01] (table 2). Normal weight diabetic patients presented obvious insulin resistance (the median of HOMA-IR value was 3.27) (table 1) and metformin treatment for 48 weeks significantly decreased HOMA-IR value by about 2.21 in normal weight group (*P*<0.05) (table 2), but acarbose did not present a similar improvement.

**Table 2:** The parameters of glucose metabolism after acarbose or metformin treatment in normal weight, overweight and obese patients with newly diagnosed type 2 diabetes.

Parameters	BMI<24		BMI 24–28		BMI>28	
	Acabose	Metfomin	Acabose	Metfomin	Acabose	Metfomin
	(n = 106)	(n = 110)	(n = 212)	(n = 193)	(n = 75)	(n = 88)
**FBG, mmol/L**						
24 weeks	−1.30 (−1.65– −0.96)	−1.82 (−2.09– −1.54)*	−1.19 (−1.41– −0.96)	−1.80 (−2.03– −1.58)*	−1.55 (−1.84– −1.25)	−1.78 (−2.09– −1.46)
48 weeks	−1.43 (−1.82– −1.06)	−1.85 (−2.22– −1.47)	−1.37 (−1.61– −1.12)	−1.73 (−1.99– −1.46)*	−1.33 (−1.71– −0.95)	−1.77 (−2.16– −1.37)
**PBG, mmol/L**						
24 weeks	−3.05 (−3.65– −2.45)	−2.79 (−3.49– −2.08)	−2.99 (−3.47– −2.51)	−2.48 (−2.95– −2.00)	−2.86 (−3.43– −2.29)	−2.59 (−3.18– −2.00)
48 weeks	−2.93 (−3.58– −2.29)	−2.35 (−3.16– −1.54)	−3.34 (−3.83– −2.84)	−2.35 (−2.85– −1.85)**	−2.40 (−3.08– −1.71)	−2.47 (−3.11– −1.82)
**FINS, uIU/mL**						
24 weeks	−2.41 (−5.61– 0.79)	−3.67 (−5.25– −2.09)	−3.79 (−5.40– −2.18)	−4.63 (−6.00– −3.25)	−4.18 (−6.89– −1.47)	−2.57 (−5.20– 0.06)
48 weeks	0.94 (0.77– 1.46)	−4.30 (−6.08– −2.52)	−3.92 (−5.74– −2.10)	−3.43 (−5.86– −1.00)	−5.57 (−8.23– −2.90)	−3.58 (−6.29– −0.87)
**HbA1C, %**						
24 weeks	−1.15 (−1.44– −0.87)	−1.17 (−1.41– −0.93)	−1.09 (−1.26– −0.92)	−1.24 (−1.42– −1.06)	−1.18 (−1.44– −0.93)	−1.30 (−1.52– −1.07)
48 weeks	−0.95 (−1.25– −0.65)	−1.10 (−1.38– −0.81)	−1.10 (−1.27– −0.93)	−1.21 (−1.42– −1.01)	−1.06 (−1.34– −0.78)	−1.21 (−1.46– −0.95)
**HbA1C≤6.5%, %**						
24 weeks	67.0	64.6	65.1	64.4	80.6	75.3
48 weeks	60.5	58.7	65.0	60.8	67.2	69.7
**HOMA-IR**						
24 weeks	−1.56 (−2.61– −0.52)	−1.88 (−2.54– −1.23)	−1.80 (−2.31– −1.29)	−2.66 (−3.28– −2.04)*	−2.41 (−3.45– −1.38)	−1.92 (−2.80– −1.04)
48 weeks	0.94 (0.77– 1.46)	−2.21 (−2.88– −1.53)	−1.90 (−2.49– −1.31)	−2.08 (−3.08– −1.08)	−2.84 (−3.90– −1.79)	−2.03 (−3.07– −0.97)
**HOMA-β**						
24 weeks	23.94 (−25.09– 72.97)	2.93 (−4.93– 10.79)	2.71 (−16.38– 21.79)	11.29 (3.07– 19.50)	16.52 (−2.14– 35.18)	20.97 (4.49– 37.44)
48 weeks	41.26 (21.23– 61.95)	4.74 (−5.32– 14.79)	9.02 (−14.80– 32.84)	7.72 (−10.32– 25.77)	2.94 (−13.34– 19.21)	12.44 (−1.09– 25.97)

Data are shown as difference (95% CI) vs baseline. FBG: fasting blood glucose; PBG: 2 h post-challenge blood glucose; FINS: fasting insulin; HbA1C: hemoglobin A1c; HOMA-IR: homeostasis model assessment of insulin resistance; HOMA-β: homeostasis model assessment of β cell function.

*significantly different at *P* <0.05 vs baseline;

**significantly different at *P* <0.01 vs baseline.

### The changes in anthropometric measurements after acarbose or metformin treatment

The BMI range of all patients was from 19 kg/m^2^ to 30 kg/m^2^ in baseline. After 24 weeks and 48 weeks treatment, both acarbose and metformin treatment resulted in a significant decrease in waist circumference, hip circumference, weight and BMI in the three groups (all *P*<0.05). The reduction of anthropometric measures was similar after acarbose or metformin treatment among normal weightand obesity groups. However, the reduction of body-weight was more in overweight patients treated with acarbose than with metformin treatment after 24 weeks and 48 weeks [24 weeks: −2.55 (−3.03 to −2.07) vs. −1.68 (−2.06 to −1.30), *P*<0.01; 48 weeks: −2.47 (−3.01 to −1.93) vs. −1.68 (−2.07 to −1.28), *P*<0.05] (table 3).

**Table 3:** The anthropometric measurements after acarbose or metformin treatment in normal weight, overweight and obese patients with newly diagnosed type 2 diabetes.

Parameters	BMI<24		BMI 24–28		BMI>28	
	Acabose (n = 106)	Metfomin (n = 110)	Acabose (n = 212)	Metfomin (n = 193)	Acabose (n = 75)	Metfomin (n = 88)
**Waist circumference, cm**						
24 weeks	−1.83 (−2.98– −0.68)	−1.64 (−2.66– −0.62)	−2.40 (−2.97– −1.82)	−2.12 (−2.79– −1.46)	−3.40 (−4.31– −2.49)	−2.28 (−3.31– −1.25)
48 weeks	−2.36 (−3.44– −1.29)	−1.81 (−2.76– −0.86)	−2.51 (−3.14– −1.88)	−2.23 (−2.92– −1.53)	−4.33 (−5.30– −3.37)	−3.03 (−4.10– −1.95)
**Hip circumference, cm**						
24 weeks	−1.52 (−2.63– −0.40)	−1.20 (−2.07– −0.33)	−2.54 (−3.21– −1.86)	−1.17 (−1.96– −0.38)*	−3.09 (−4.13– −2.05)	−2.41 (−3.54– −1.29)
48 weeks	−2.13 (−3.09– −1.18)	−1.64 (−2.46– −0.81)	−2.53 (−3.21– −1.85)	−1.75 (−2.53– −0.97)	−3.57 (−4.85– −2.29)	−2.72 (−3.69– −1.76)
**Weight, kg**						
24 weeks	−1.78 (−2.38– −1.18)	−1.40 (−2.00– −0.81)	−2.55 (−3.03– −2.07)	−1.68 (−2.06– −1.30)**	−3.55 (−4.44– −2.66)	−2.84 (−3.64– −2.04)
48 weeks	−1.44 (−2.14– −0.75)	−1.43 (−2.06– −0.80)	−2.47 (−3.01– −1.93)	−1.68 (−2.07– −1.28)*	−4.00 (−5.05– −2.95)	−2.95 (−3.73– −2.17)
**BMI, kg/m^2^**						
24 weeks	−0.78 (−1.03– −0.53)	−0.43 (−0.70– −0.15)	−1.09 (−1.26– −0.92)	−0.79 (−0.96– −0.63)*	−1.51 (−1.86– −1.16)	−1.32 (−1.63– −1.02)
48 weeks	−0.63 (−0.91– −0.35)	−0.45 (−0.74– −0.15)	−1.07 (−1.28– −0.86)	−0.81 (−0.97– −0.65)	−1.67 (−2.07– −1.28)	−1.37 (−1.67– −1.07)

Data are shown as difference (95% CI) vs baseline. BMI: body mass index.

*significantly different at *P*<0.05 vs baseline;

**significantly different at *P*<0.01 vs baseline.

### Effect of acarbose and metformin treatment on lipid profile and blood pressure

A significant decline of plasma TC and non-HDL-C was observed in the three groups after acarbose and metformin treatment for 24 weeks and 48 weeks (all *P*<0.05). Acarbose decreased plasma level of TG significantly as compared with metformin both in overweight and obesity groups [24 weeks: −0.47 (−0.85 to −0.09) vs. 0.15 (−0.15 to 0.45), *P*<0.05; 48 weeks: −0.48 (−0.74 to −0.21) vs. 0.20 (−0.289 to 0.70), *P*<0.05]. Moreover, we evaluated the proportion of patients with optimal levels of LDL-C and non-HDL-C. The optimal levels of LDL-C and non-HDL-C were defined as: LDL-C<100 mg/dL (2.6 mmol/L) and non-HDL-C<130 mg/dL (2.6 mmol/L) [8] Interestingly, the proportion of patients with optimal LDL-C levels was higher in obese patients treated with metformin than acarbose (table 4) [24 weeks: 43.9% vs. 28.6%, *P*<0.05; 48 weeks: 42.1% vs. 19.7%, *P*<0.01].

**Table 4:** The changes of lipid profile and blood pressure after acarbose or metformin treatment in normal weight, overweight and obese patients with newly diagnosed type 2 diabetes.

Parameters	BMI<24		BMI 24–28		BMI>28	
	Acabose (n = 106)	Metfomin (n = 110)	Acabose (n = 212)	Metfomin (n = 193)	Acabose (n = 75)	Metfomin (n = 88)
**Systolic BP, mm Hg**						
24 weeks	−0.03 (−2.26– 2.20)	−1.58 (−4.10– 0.93)	−2.50 (−4.23– −0.77)	−1.96 (−4.10– 0.19)	0.54 (−3.02– 4.11)	−0.67 (−3.28– 1.93)
48 weeks	0.64 (−1.74– 3.02)	0.45 (−1.90– 2.81)	−1.40 (−3.31– 0.50)	−2.00 (−4.03– 0.03)	1.19 (−2.01– 4.39)	−1.47 (−4.33– 1.40)
**Diastolic BP, mm Hg**						
24 weeks	−1.37 (−3.00– 0.27)	−1.26 (−3.04– 0.52)	−2.45 (−3.77– −1.14)	−1.34 (−2.81– 0.14)	−1.73 (−4.35– 0.89)	−2.06 (−4.18– 0.06)
48 weeks	−0.16 (−2.08– 1.75)	−1.10 (−2.68– 0.49)	−2.24 (−3.53– −0.96)	−1.29 (−2.73– 0.14)	−3.16 (−5.90– −0.42)	−2.68 (−4.93– −0.42)
**TC, mmol/L**						
24 weeks	−0.30 (−0.49– −0.11)	−0.26 (−0.46 − −0.06)	−0.42 (−0.57– −0.27)	−0.33 (−0.48 − −0.17)	−0.49 (−0.71– −0.26)	−0.51 (−0.73 − −0.29)
48 weeks	−0.19 (−0.41– 0.02)	−0.22 (−0.41 − −0.03)	−0.39 (−0.54– −0.23)	−0.34 (−0.52 − −0.16)	−0.45 (−0.70– −0.20)	−0.46 (−0.71 − −0.22)
**LDL-C, mmol/L**						
24 weeks	−0.19 (−0.38– −0.01)	−0.17 (−0.33 − −0.02)	−0.15 (−0.27– −0.02)	−0.21 (−0.34 − −0.08)	−0.16 (−0.35– 0.03)	−0.35 (−0.52 − −0.19)
48 weeks	−0.07 (−0.27– 0.13)	−0.09 (−0.23 − −0.06)	−0.09 (−0.21– 0.04)	−0.13 (−0.28 − 0.02)	0.02 (−0.20– 0.24)	−0.20 (−0.39 − −0.01)
**HDL-C, mmol/L**						
24 weeks	0.07 (0.00– 0.13)	0.03 (−0.05– 0.11)	0.00 (−0.03– 0.03)	−0.04 (−0.09– 0.00)	0.07 (0.01– 0.13)	0.03 (−0.02– 0.08)
48 weeks	0.06 (−0.01– 0.12)	0.00 (−0.09– 0.08)	0.01 (−0.03– 0.05)	−0.03 (−0.07– 0.02)	0.01 (−0.04– 0.07)	−0.03 (−0.09– 0.03)
**TG, mmol/L**						
24 weeks	−0.32 (−0.87– 0.24)	0.26 (−0.33– 0.86)	−0.47 (−0.85– −0.09)	0.15 (−0.15– 0.45)*	−0.94 (−1.36– −0.52)	−0.13 (−0.48– 0.21)**
48 weeks	−0.29 (−0.56– −0.02)	−2.13 (−3.09 − −1.18)	−0.48 (−0.74– −0.21)	0.20 (−0.289– 0.70)*	−0.93 (−1.40– −0.45)	−0.17 (−0.67 − −0.32)*
**Non-HDL-C, mmol/L**						
24 weeks	−0.36 (−0.56– −0.17)	−0.27 (−0.46 − −0.07)	−0.42 (−0.58– −0.27)	−0.29 (−0.44 − −0.13)	−0.56 (−0.77– −0.34)	−0.54 (−0.76 − −0.33)
48 weeks	−0.25 (−0.46– −0.05)	−0.21 (−0.39 − −0.03)	−0.40 (−0.55– −0.25)	−0.31 (−0.49 − −0.13)	−0.47 (−0.70– −0.23)	−0.44 (−0.69 − −0.19)
**optimal LDL**−**C rate, %**						
24 weeks	43.5	34.4	30.5	40.2	28.6	43.9*
48 weeks	39.5	36.6	32.8	32.5	19.7	42.1**
**optimal non-HDL-C rate, %**						
24 weeks	51.1	45.8	41.4	42.7	38.6	45.1
48 weeks	44.2	44.1	42.1	40.8	45.5	42.1

Data are shown as difference (95% CI) vs baseline, unless optimal LDL-C rate and optimal non-HDL-C rate. BP: blood pressure; TC: total cholesterol; LDL-C: low-density lipoprotein cholesterol; HDL-C: high-density lipoprotein cholesterol; TG: triglyceride; FBG: fasting blood glucose; optimal LDL-C rate: the proportion of patients with optimal levels of LDL-C; optimal non-HDL-C rate: the proportion of patients with optimal levels of non-HDL-C. The optimal levels of LDL-C and non-HDL-C were defined as: LDL-C<100 mg/dL (2.6 mmol/L) and non-HDL-C<130 mg/dL (2.6 mmol/L) [7].

*significantly different at *P*<0.05 vs baseline;

**significantly different at *P*<0.01 vs baseline.

Acarbose reduced diastolic blood pressure by about 2.2–3.2 mmHg in overweight and obese patients (*P*<0.05), and metformin decreased diastolic blood pressure of obese patients by about 2.7 mmHg (*P*<0.05). No significant difference of systolic blood pressure was observed among the three groups (table 4).

## Discussion

Chinese, other than people in the western country, have certain genetic backgrounds and favor high carbohydrate diet. Acarbose binds with α-glucosidases in the brush border of the small intestine and reduces the rate of carbohydrate absorption [12]. So it is thought to have low capability of glucose-lowering effect and be more suitable for people who like high carbohydrate diet [12]. Nevertheless, the recent result of MARCH trial demonstrates that acarbose and metformin have similar efficacy asinitial therapy for HbA1c reduction in newly diagnosed type 2 diabetic patients in China [6]. However, it was still unclear whether the above mentioned therapeutic efficacy was diversified under the pathological condition of obesity. Our present study showed that the reduction of HbA1c levels and the proportion of patients with HbA1c of 6.5% or less were similar in different BMI status after acarbose and metformin treatment. So the glucose-decreasing effect of acarbose was similar to metformin in Chinese patients newly diagnosed with type 2 diabetes regardless of patient's weight. Only in overweight patients, metformin showed more therapeutic effect on FBG levels, and acarbose exerted better effect on PBG levels.

Obesity is associated with insulin resistance [1]. In the present study, Chinese patients with newly diagnosed type 2 diabetes had a greater percentage of overweight. Besides, the mean ages of overweight and obese diabetic patients were younger than normal weight patients. As body weight and BMI increased, insulin resistance became more pronounced and the prevalences of hypertension and non-alcoholic fatty liver disease also got higher. The impact on weight is one of important aspects for evaluating the clinical value of hypoglycemic drugs. Our study demonstrated that acarbose and metformin respectively leaded to a significant and modest decrease of body-weight in different BMI status, and greater weight loss corresponded to higher BMI. Interestingly, acarbose showed a slimilar weight-loss effect with metformin. Our results about metformin were consistent with others [13]–[14]. But acarbose only results in slight weight loss of diabetic patients in other previous studies [15]. The difference of weight loss about acarbose may be related to high carbohydrate diet habits and genetic backgrounds of Chinese people. As the α-glucosidase inhibitor, acarbose shows an obvious advantage to Chinese diabetic patients. Acarbose causes weight loss by inhibition of carbohydrate digestion and delayed gastric emptying, but does not seem to have a significant effect on nutrient intake [16]. Metformin's contribution to weight loss may be explained through the reduction of carbohydrates absorption and ghrelin levels after glucose overload, the induction of a lipolitic and anoretic effect, and the improvement of insulin sensitivity [17]–[18].

Insulin resistance plays a critical mechanism in the pathogenesis of type 2 diabetes and related complication [19]. Metformin is widely believed to be a stronger insulin sensitizer than acarbose, especially in obese patients [20]. Metformin improves insulin sensitivity indirectly by reducing weight and regulating lipid metabolism, and also directly by stimulating insulin signaling pathway and the expression of insulin receptors and GLUT4 [21]–[22]. Our present study showed that acarbose and metformin have similar therapeutic efficacy on insulin resistance in overweight and obese diabetic patients. High carbohydrate diet makes Chinese prone to postprandial hyperglycemia, which is the main feature of newly diagnosed type 2 diabetic patients. Postprandial hyperglycemia causes insulin resistance by many mechanisms, including enhancing advanced glycation end products and oxidative stress, promoting production of inflammatory factors and disturbing insulin signaling pathway [23]–[24]. Thus, acarbose may improve insulin sensitivity by reducing postprandial blood glucose levels [23], and the decrease of weight maybe also associated with the insulin sensitizing effect of acarbose. It's worth noting that normal weight diabetic patients presented obvious insulin resistance and metformin significantly ameliorated insulin resistance of normal weight diabetic patients, but acarbose did not present a similar improvement.

In the aspect of improvement on components of metabolic syndrome, acarbose and metformin also revealed some beneficial effects. Our study was consistent with others [25]–[28] and showed that acarbose and metformin treatment causes a slight decline of plasma TC and non-HDL-C in the three BMI groups. Acarbose decreased TG more intensively as compared with metformin in overweight and obesity groups. The proportion of patients with optimal LDL-C levels with metformin treatment was higher than acarbose in obese patients. Acarbose has a beneficial effect on lipid profile through multiple mechanisms, including the reduction of plasma glucose levels, insulin resistance and body weight [29]. The several clinical trials have shown the blood pressure lowering effects of acarbose and metformin [30]–[33]. In the present study, acarbose and metformin decreased diastolic blood pressure of obese patients, and had no effect on systolic blood pressure. Insulin resistance may contribute to hypertension by increasing activity of sympathetic, renal sodium retention and vascular smooth muscle tone and proliferation [34], so the effect of acarbose and metformin on blood pressure could be related to decreased body weight and insulin resistance. Except through glucose-lowering and insulin resistance-improving, metformin has a direct effect on lipid metabolism by inhibiting mitochondrial complex I and promoting AMPK-dependent catabolic pathways [35]–[36]. The activation of AMPK inactivates acetyl-CoA carboxylase and 3-hydroxy-3-methylglutaryl (HMG)-CoA reductase, inhibits the sterol regulatory element-binding protein-1c (SREBP-1c), which results in a decrease of fatty acid and cholesterol synthesis [37].

## Conclusion

Acarbose and metformin decreased HbA1c levels similarly regardless of BMI status of Chinese patients with newly diagnosed type 2 diabetes. Acarbose and metformin resulted in a significant and modest improvement of anthropometric parametres in different BMI status. Thus, our results provide new clinical evidence to support the idea that acarbose treatment may contribute a similar effect to plasma glucose control compared to metformin in Chinese diabetic patients, even in obesity patients.
